# Silicon Foliar Application Mitigates Salt Stress in Sweet Pepper Plants by Enhancing Water Status, Photosynthesis, Antioxidant Enzyme Activity and Fruit Yield

**DOI:** 10.3390/plants9060733

**Published:** 2020-06-10

**Authors:** Khaled A. A. Abdelaal, Yasser S.A. Mazrou, Yaser M. Hafez

**Affiliations:** 1 Plant Pathology and Biotechnology Lab., Excellence Center (EPCRS), Faculty of Agriculture, Kafrelsheikh University, Kafr Elsheikh 33516, Egypt; 2 Business Administration Department, Community College, King Khalid University, Guraiger, Abha 62529, Saudi Arabia; ymazrou@kku.edu.sa; 3 Faculty of Agriculture, Tanta University, Tanta 31512, Egypt; 4 Excellence Center (EPCRS), Plant Pathology and Biotechnology Lab, Faculty of Agriculture, Kafrelsheikh University, Kafr Elsheikh 33516, Egypt; hafezyasser@gmail.com

**Keywords:** *Capsicum annuum* L., silicon, salinity, chlorophyll, lipid peroxidation, reactive oxygen species

## Abstract

Silicon is one of the most significant elements in plants under abiotic stress, so we investigated the role of silicon in alleviation of the detrimental effects of salinity at two concentrations (1500 and 3000 ppm sodium chloride) in sweet pepper plants in two seasons (2018 and 2019). Our results indicated that relative water content, concentrations of chlorophyll a and b, nitrogen, phosphorus and potassium contents, number of fruits plant^−1^, fruit fresh weight plant^−1^ (g) and fruit yield (ton hectare^−1^) significantly decreased in salt-stressed sweet pepper plants as compared to control plants. In addition, electrolyte leakage, proline, lipid peroxidation, superoxide (O_2_^−^) and hydrogen peroxide (H_2_O_2_) levels, soluble sugars, sucrose, and starch content as well as sodium content significantly increased under salinity conditions. Conversely, foliar application of silicon led to improvements in concentrations of chlorophyll a and b and mineral nutrients, water status, and fruit yield of sweet pepper plants. Furthermore, lipid peroxidation, electrolyte leakage, levels of superoxide, and hydrogen peroxide were decreased with silicon treatments.

## 1. Introduction

Sweet pepper is one of the most significant vegetable crops, belongs to the Solanaceae family, and has a high importance in the exporting market. About 14% of sweet pepper fruit loss is attributed to increased salt levels [1]. Salinity is one of the major restricting abiotic factors for agricultural production worldwide [2], affecting osmotic adjustment and solutes uptake resulting in many harmful changes in morphological and physiological characters such as photosynthetic rate and plasma membrane permeability of many economic plants [3,4,5,6]. Morphological traits such as leaf number and leaf area can be negatively affected under salinity stress [7]. Furthermore, physiological and biochemical characters can be adversely affected under salinity stress conditions, e.g., decreases in chlorophyll concentrations and relative water contents, reduced essential nutrient uptake as well as increased electrolyte leakage may occur [8]. In addition, accumulation of reactive oxygen species (ROS) such as superoxide (O_2_^−^) and hydrogen peroxide (H_2_O_2_) may increase, causing oxidative stress [9] as well as delays in cellular metabolism as one of the important signals of many stresses such as salinity [10], drought [11,12,13], chilling [14] and biotic stress [15] in several economically important crop plants. The high concentration of reactive oxygen species reduces nutrient uptake, damages proteins, and lipids as well as metabolic pathways [16]. On the other hand, enzymatic antioxidants (e.g., catalase, peroxidase, and superoxide dismutase) and non-enzymatic antioxidants (ascorbic acid and carotene) are accumulated under various stresses to deal with the increase of ROS and protect the plant tissues from oxidative damage under various stresses [17,18]. Therefore, plants need several strategies to overcome the detrimental effects of salt stress through osmotic adjustments and photosynthetic process improvements [19].

Silicon (Si) and salicylic acid were used to mitigate the injurious effects of various stresses which affect growth traits and production. Salicylic acid application led to improvements in plant height, number of leaves, leaf area, concentrations of chlorophyll, and grain yield of fava bean plants under drought conditions [11] and under salinity stress conditions [10]. Silicon is one of the most important elements, covering about 28% of the lithosphere and recently involved as a “quasi-essential” element according to The International Plant Nutrition Institute. Application of silicon is an important method to minimize the adverse impacts of salinity stress, particularly in sunflower [20] and sorghum [21] causing enhancement of morphophysiological growth traits, for example, plant height and concentrations of chlorophyll in *Salvia splendens* and tomato plants under salinity stress conditions [22,23]. Moreover, plants can uptake silicon in the form of soluble silicic acid at pH ≤ 9 [24]. Silicon can alleviate several types of abiotic stresses such as salinity, drought, and low temperature [25,26]. Silicon treatments led to maintenance of water status through regulating transpiration rates and formation of a double cuticle layer on the epidermis of leaves [18] resulting in a decreased transpiration and increased photosynthetic rate. In addition, Si application improves stomatal conductance and carbon dioxide assimilation rate in sorghum plants under salinity stress conditions [27] that is likely due to the increases of number and size of stomata as shown in okra plants. Exogenous application of silicon improved the injurious effects of salinity stress on stomatal movement of sweet pepper (*Capsicum annuum*) plants [28] and enhanced the expression of a zinc finger protein that regulates stomatal movement in rice plants under salinity stress [29]. Silicon treatment under salinity stress led to protection of photosynthetic pigments from degradation and regulation of photosynthesis-related proteins [30]. Generally, the role of silicon in stress tolerance mechanisms can be explained by improvements in growth character, increased nutrient uptake, and photosynthetic rate as well as antioxidant defense system activation [18].

Silicon can be regarded as one of the most important elements in agricultural production, particularly in justification of salinity stress effects, which—as one of the main abiotic stresses—causes detrimental impacts for most plants grown in various global regions. However, the information on the effects of silicon on sweet pepper plants under salinity is still insufficient. Thus the aim of our study was to evaluate the effects of foliar application of silicon on concentrations of chlorophyll, relative water content, lipid peroxidation, proline contents, up-regulation of antioxidant enzymes, reactive oxygen species and mineral contents as well as fruit yield of sweet pepper under salinity stress conditions.

## 2. Results

### 2.1. Effects of Silicon on Concentrations of Chlorophyll a and b, Relative Water Content (RWC %), and Proline in Salt-Stressed Sweet Pepper Plants

**Figure 1:**
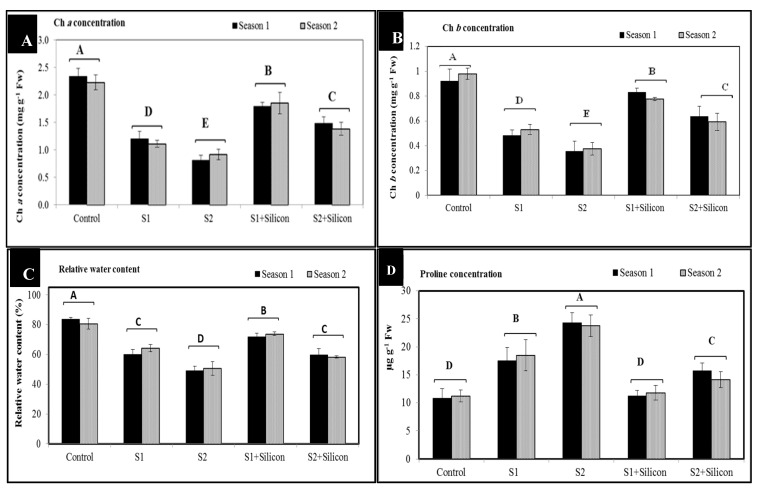
Effects of silicon on concentrations of chlorophyll a (**A**), chlorophyll b (**B**), relative water content (**C**), and proline (**D**) in sweet pepper plants under salinity stress in the 2018 and 2019 seasons. Bars followed by different letters are significantly different according to Duncan’s multiple range tests (DMRTs) at *p* < 0.05. S1: Salinity at 1500 ppm, S2: Salinity at 3000 ppm.

It can be noted from Figure 1 that the concentrations of chlorophyll a (1.2 and 0.8 mg g^−1^ Fw) and b (0.5 and 0.35 mg g^−1^ Fw) were significantly decreased in sweet pepper plants under both levels of salinity (1500 and 3000 ppm) respectively, as compared to controls (chlorophyll a 2.2 and chlorophyll b 0.92 mg g^−1^ Fw). In addition, relative water content significantly decreased (62 and 50%) at the two salinity levels (1500 and 3000 ppm) compared to controls (82%), while proline concentrations (18 and 24 μg g^−1^ Fw) significantly increased in sweet pepper plants under salinity conditions in the mean of both seasons as compared to controls (11 μg g^−1^ Fw). However, application of silicon significantly increased concentrations of chlorophyll a (1.9 and 1.5 mg g^−1^ Fw) as compared to untreated, salt-stressed plants (1.2 and 0.8 mg g^−1^ Fw) at 1500 and 3000 ppm respectively. Additionally, chlorophyll b significantly increased (0.82 and 0.63 mg g^−1^ Fw) compared to untreated plants (0.48 and 0.35 mg g^−1^ Fw) at 1500 and 3000 ppm salt stress. Silicon application led to significant increases in relative water contents (71 and 59%) as compared to untreated sweet pepper plants (60 and 49%) at 1500 and 3000 ppm respectively. Proline accumulation under silicon treatment was 11.5 and 14.4 μg g^−1^ Fw in salt-stressed plants at 1500 and 3000 ppm.

### 2.2. Effects of Silicon on Electrolyte Leakage (EL %), Lipid Peroxidation (MDA), Levels of Superoxide, and Hydrogen Peroxide in Sweet Pepper Under Salinity Stress

**Figure 2:**
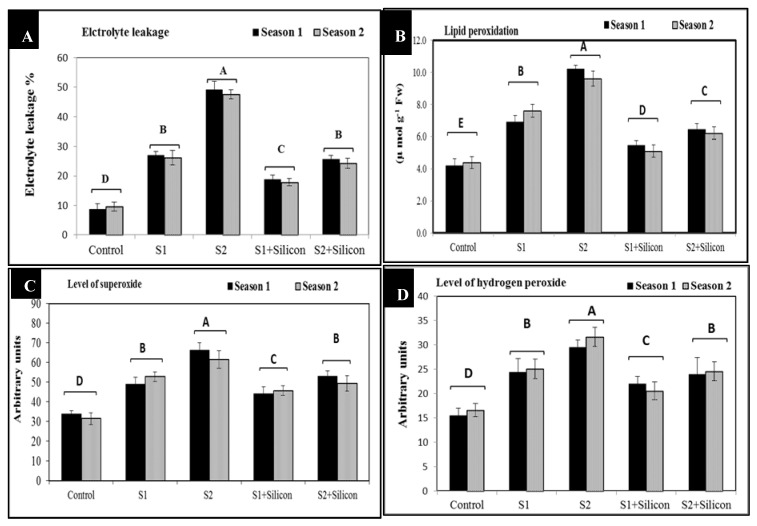
Effects of silicon on electrolyte leakage (EL %) (**A**), lipid peroxidation (MDA) (**B**), levels of superoxide (**C**) and hydrogen peroxide (**D**) in salt-stressed sweet pepper plants in the 2018 and 2019 seasons. S1: Salinity at 1500 ppm, S2: Salinity at 3000 ppm.

The obtained results in Figure 2 showed that foliar application of silicon considerably reduced electrolyte leakage (EL %), lipid peroxidation (MDA), levels of superoxide and hydrogen peroxide in salt-stressed sweet pepper plants in both seasons. Electrolyte leakage (%) significantly decreased (18% and 25%) under application of silicon in both salinity levels (1500 and 3000 ppm) as compared to the untreated stressed plants (27% and 49%) as the mean of two seasons. However, electrolyte leakage significantly increased (27% and 49%) in the stressed untreated plants when compared to control plants (9%). In addition, salinity stress at 1500 and 3000 ppm elicited a significant increase in lipid peroxidation (7.2 and 9.8 μmol/g Fw) (malondialdehyde) in sweet pepper plants at two levels of salinity as compared to control plants (4.3 μmol/g Fw). The higher level of salinity (3000 ppm) caused the highest levels of malondialdehyde. Interestingly, silicon application led to a significant decrease in malondialdehyde (5.3 and 6.2 μmol/g Fw) in the stressed plants compared to the stressed untreated plants (7.2 and 9.8 μmol/g Fw).

The levels of superoxide and hydrogen peroxide significantly increased under salinity stress conditions (1500 and 3000 ppm), the higher level of salinity (3000 ppm) produced the highest values of superoxide (64 arbitrary units) and hydrogen peroxide (24.5 arbitrary units) as the mean of both seasons. Nevertheless, the silicon-treated salt-stressed sweet pepper plants showed significant decreases in superoxide (45 and 51.5 arbitrary units) as compared to the stressed untreated plants (51 and 63 arbitrary units). Additionally, hydrogen peroxide levels were decreased significantly (21.5 and 24.3 arbitrary units) under foliar application of silicon compared to the stressed untreated plants (24.7 and 30.5 arbitrary units) at both levels of salinity (1500 and 3000 ppm).

### 2.3. Effects of Silicon on the Activity of Antioxidant Enzymes (CAT, POX, and GR) and PPO in Salt-Stressed Sweet Pepper Plants

**Figure 3:**
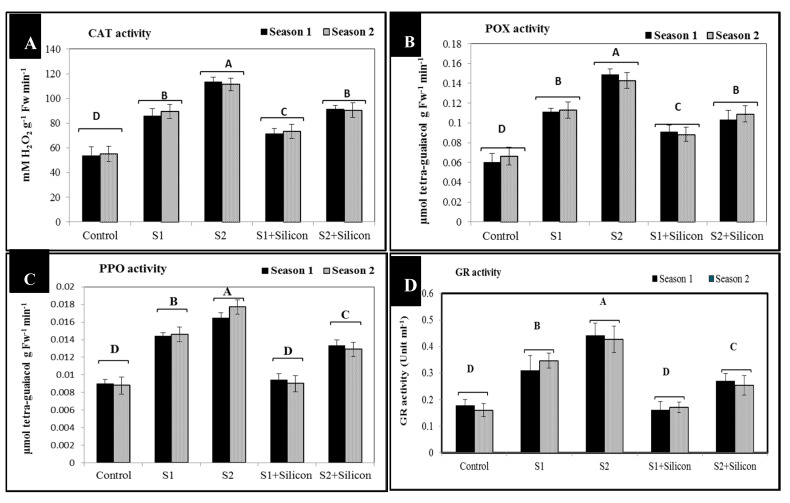
Activities of catalase (CAT) (**A**), peroxidase (POX) (**B**), polyphenol oxidase (PPO) (**C**) and glutathione reductase (GR) (**D**) as affected by silicon in salt-stressed sweet pepper plants in the 2018 and 2019 seasons. S1: Salinity at 1500 ppm, S2: Salinity at 3000 ppm.

The presented results in Figure 3 show that the antioxidant enzyme activity (catalase (CAT), peroxidase (POX), and glutathione reductase (GR)) and polyphenol oxidase (PPO) activity significantly increased in plants exposed to salinity stress at two concentrations (1500 and 3000 ppm) in both seasons. Activity of catalase significantly increased in salt-stressed plants (87 and 112 mM H_2_O_2_ g^−1^ Fw min^−1^), as compared to salt-stressed and silicone-treated plants (90.8 and 72) and controls (54.4). Additionally, peroxidase significantly increased in salt-stressed plants (0.11 and 0.14 μmol tetra-guaiacol g Fw^−1^ min^−1^) as compared to stressed and silicone-treated plants (0.09 and 0.10 mM H_2_O_2_ g^−1^ Fw min^−1^) and controls (0.62). Moreover, polyphenol oxidase activity significantly increased in salt-stressed untreated plants (0.11 and 0.16 μmol tetra-guaiacol g Fw^−1^ min^−1^) as compared to stressed, silicon-treated plants (0.009 and 0.013 μmol tetra-guaiacol g Fw^−1^ min^−1^) and controls (0.008). Activity of glutathione reductase significantly increased in stressed untreated plants (0.32 and 0.43 unit ml^−1^) and in the treated plants (0.17 and 0.26 unit ml^−1^) as compared to control plants (0.16). Foliar application of silicon successfully up-regulated the activity of catalase, peroxidase, polyphenol oxidase, and glutathione reductase in sweet pepper plants under salinity conditions (Figure 3A–D).

### 2.4. Effects of Silicon on Soluble Sugars, Starch Contents, and Sucrose in Sweet Pepper Plants Under Salinity Conditions

**Figure 4:**
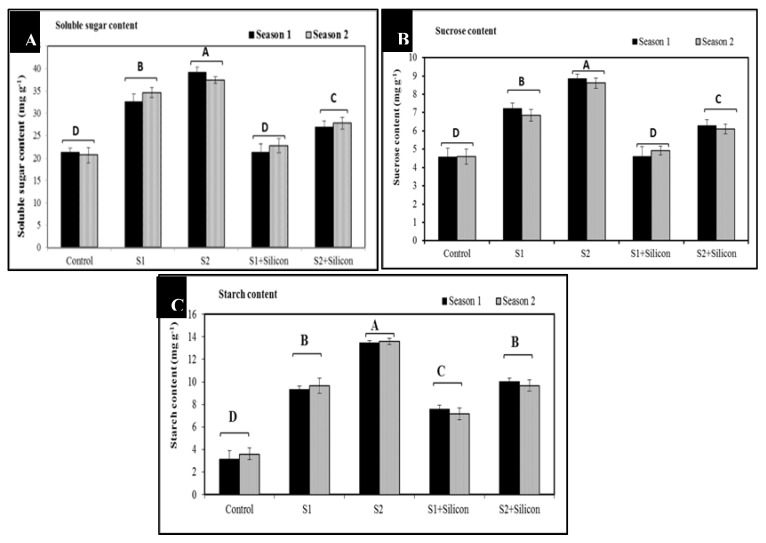
Effects of silicon on soluble sugar (**A**), sucrose (**B**) and starch contents (**C**) in salt-stressed sweet pepper plants in the 2018 and 2019 seasons. S1: Salinity at 1500 ppm, S2: Salinity at 3000 ppm.

Our findings in Figure 4 indicated that soluble sugars, as osmolytes, significantly increased in sweet pepper plants as a response to salinity stress (33.5 and 38.1 mg g^−1^) and in the stressed plants treated with silicon (22 and 27.2 mg g^−1^) at the two concentrations of salinity (1500 and 3000 ppm) as compared to control plants (21 mg g^−1^). Additionally, sucrose (7 and 8.7 mg g^−1^) and starch contents (9.5 and 13.4 mg g^−1^) obviously increased in the sweet pepper plants under the two concentrations of salinity as compared to control plants (4.5 and 3.4 mg g^−1^) in the mean of both seasons. In addition, the stressed and silicone-treated plants showed significant increases in sucrose (4.8 and 6.2 mg g^−1^) and in starch contents (7.4 and 8.8 mg g^−1^) as compared to control plants (4.5 and 3.4 mg g^−1^).

### 2.5. Effects of Silicon on Nitrogen, Phosphorus, Potassium, and Sodium Contents in Salt-Stressed Sweet Pepper Plants

**Figure 5:**
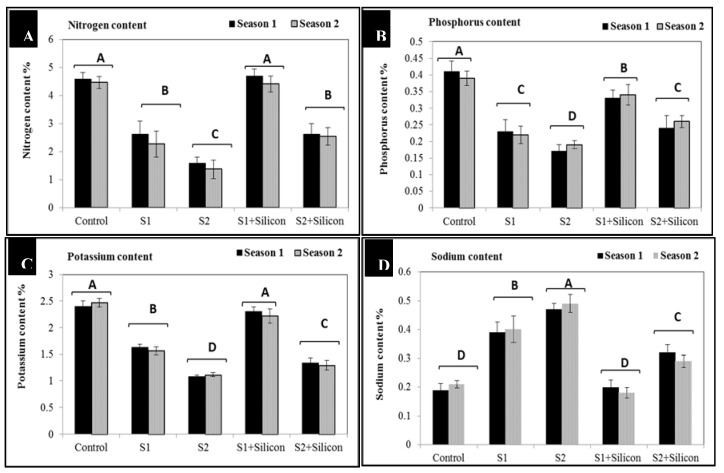
Effects of silicon on nitrogen (**A**), phosphorus (**B**), potassium (**C**), and sodium (**D**) contents in salt-stressed sweet pepper plants in the 2018 and 2019 seasons. S1: Salinity at 1500 ppm, S2: Salinity at 3000 ppm.

The obtained results in Figure 5 indicated that the salinity-exposed sweet pepper plants show significant decreases in nitrogen (2.4% and 1.4%), phosphorus (0.23% and 0.18%), and potassium contents (1.6% and 1.1%) as compared to the control treatments (4.5%, 0.4% and 2.4%) respectively. Nonetheless, silicon application significantly increased the content of nitrogen (4.5% and 2.6%), phosphorus (0.33% and 0.25%) and potassium (2.25% and 1.3%) in stressed sweet pepper plants at both concentrations (1500 and 3000 ppm) of salinity. Silicon application gives the best results in increasing nitrogen and potassium contents under low salinity (1500 ppm) but without significant differences when compared to control plants. On the contrary, sodium contents significantly increased in the salt-stressed plants (0.4% and 0.48%) under the two salinity levels (1500 and 3000 ppm), the higher sodium content (0.48%) was observed under the higher level of salinity (3000 ppm). However, sweet pepper plants pretreated with foliar applications of silicon showed sodium contents (0.19% and 0.30%) much lower than in the stressed untreated plants (0.4% and 0.48%).

### 2.6. Effects of Silicon on Numbers of Fruits Plant^−1^, Fresh Weight Of Fruits Plant^−1^, and Fruit Yield (Ton Hectare^−1^)

**Figure 6:**
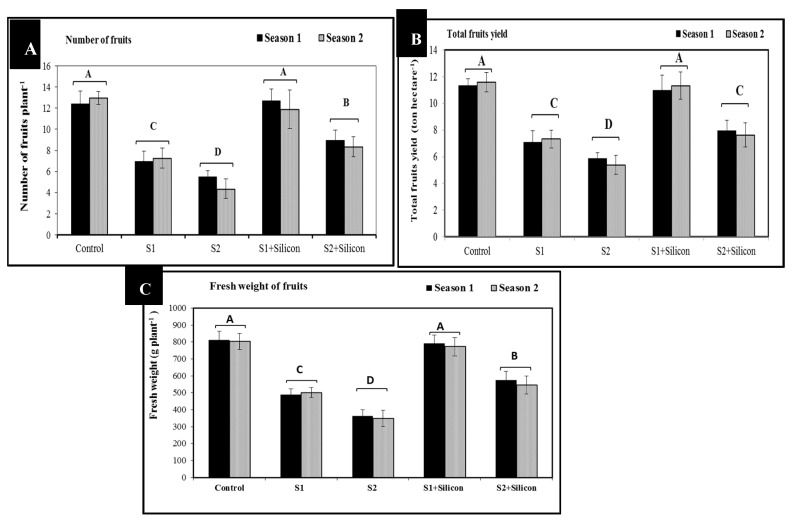
Effects of silicon on number of fruits plant^−1^ (**A**), total fruit yield (ton hectare^−1^) (**B**) and fruit fresh weight plant^−1^ (**C**) in salt-stressed sweet pepper plants in the 2018 and 2019 seasons. S1: Salinity at 1500 ppm, S2: Salinity at 3000 ppm.

The presented results in Figure 6 revealed that salinity stress led to significant decreases in number of fruits plant^−1^ (7.1 and 5.4), total fruit yield (7.1 and 5.4 ton hectare^−1^) and fruit fresh weight plant^−1^ (495 and 355 g) in the sweet pepper plants exposed to salinity stress as compared to the control treatments (12.5, 11.5 ton and 807 g) respectively. Moreover, foliar application of silicon caused significant increases in number of fruits plant^−1^ (12.8 and 8.6), total fruit yield (ton hectare-1) (11.1 and 7.8 ton), and fruit fresh weight plant^−1^ (781 and 560 g) in the stressed sweet pepper plants under the two salinity levels. Under silicon treatment, the best results of number of fruits plant^−1^ (12.8), total fruit yield (11.1 ton) and fruit fresh weight plant^−1^ (781 g) in the stressed plants were obtained at the lower level of salinity (1500 ppm). These increases were significant when compared to stressed untreated plants pointing to the positive role of silicon.

## 3. Discussion

Salt stress adversely affects the growth characters and differentiation of sweet pepper. The harmful effects of salinity on concentrations of chlorophyll a and b as well as relative water content of sweet pepper plants as compared to the control as presented in Figure 1 could be attributed to the role of salinity in inhibition of ribulose-1,5-biphosphate, increased chloroplast degradation and chloroplast structure disorder, consequently, decreased chlorophyll concentrations. In addition, relative water content significantly decreased as a result of the negative effect of salinity on water and absorption capacity from the soil to the root system, reducing the internal water supply and negatively affecting plant organs [10,31]. Proline content was also increased in sweet pepper under salinity stress conditions. This increment of proline is one of the typical responses to stress factors, particularly salinity stress, which plays a pivotal role in protecting chlorophyll pigments from degradation [27]. Besides its role as an osmolyte, proline also plays a central role in scavenging reactive oxygen species (ROS), controlling cell redox homeostasis and supplying energy [32]. Nevertheless, the significant increases in concentrations of chlorophyll a and b as well as relative water content in salt-stressed and silicon-treated sweet pepper plants could be due to the helpful role of silicon in reducing Na^+^ uptake and increasing K^+^ concentrations and uptake [33] which improves activities of several enzymes, promotes photosynthesis and water status as well as relative water content, and inhibits chlorophyll degradation. Consistent with our findings, it has been stated that application of silicon increases potassium uptake and decreases Na^+^ under salinity and drought stress conditions in many plants [34,35,36,37].

In the current work, we investigated the impact of silicon on salt-stressed sweet pepper plants. Electrolyte leakage (EL), lipid peroxidation, levels of superoxide, and hydrogen peroxide are important signals of salinity stress (Figure 2). These parameters significantly increased under the two salinity levels, this result could be due to the deleterious effect of salinity on sweet pepper plants, the damaging effect on membrane stability and selective permeability, consequently increasing electrolyte leakage [38]. Moreover, lipid peroxidation, assayed as malondialdehyde (MDA) accumulation, significantly increased as a stress factor under salinity stress conditions, this increase in MDA might be due to the oxidative damage to chloroplasts and mitochondria, subsequently increasing lipid peroxidation. Additionally, reactive oxygen species, mainly hydrogen peroxide and superoxide, have considerably increased in salt-stressed sweet pepper plants. The accumulation of superoxide and hydrogen peroxide under salinity stress at two levels (1500 and 3000 ppm) is an important indicator of oxidative stress and plays a pivotal role in regulating the development and differentiation as well as stress signaling in plant organs [35,36,37]. Furthermore, silicon application alleviates the harmful effects of salinity including decreases in electrolyte leakage, lipid peroxidation, levels of superoxide, and hydrogen peroxide. This may be due to the fact that silicon has an important role in regulating plasma membrane stability and increasing osmolyte accumulation which results in scavenging of reactive oxygen species, mainly hydrogen peroxide and superoxide [37].

The activity of the antioxidant enzymes catalase, peroxidase, and glutathione reductase and that of polyphenol oxidase significantly boosted in the sweet pepper plants exposed to salinity stress in both seasons (Figure 3). The increases of these enzyme activities under salt stress are very important in dealing with the adverse effects of salinity by scavenging reactive oxygen species. Our findings are in line with the obtained results of researchers investigating several plant species under different types of stresses [38,39]. In the current study, we have shown that exogenous application of silicon regulates antioxidant enzyme activities and maintains reactive oxygen species levels at nontoxic concentrations, protecting sweet pepper plants against oxidative damage under salinity conditions [40]. The valuable influence of silicon in enhancing the activities of catalase, peroxidase, polyphenol oxidase, and glutathione reductase enzymes as well as the elimination of superoxide and hydrogen peroxide could be due to the effect of silicon in controlling oxidative stress by alleviating ion toxicity and accumulation of nucleoproteins that contribute to plant resistance to stress factors [41].

The significant increases in soluble sugars, sucrose, and starch contents in sweet pepper under salinity stress (Figure 4) may be due to the above-mentioned traits. These compounds play pivotal roles as osmolytes and osmoprotectants in regulation of osmotic pressure under salinity stress conditions. Furthermore, adding silicon to sweet pepper plants under salinity conditions led to improved accumulation of these osmolytes, subsequently enhancing the tolerance of sweet pepper to salinity stress through biosynthetic regulation of osmolytes and some plant hormones. The positive effect of osmolytes on stressed sweet pepper plants could be attributed to the role of osmolytes in membrane stability and preventing the physiological drought of plant cells [42]. A similar trend with accumulation of osmolytes under various stresses was recorded [43,44,45,46]. The detrimental effects of salinity could be due to its adverse effect on nutrient uptake by down-regulation of some proteins such as NRT1 and AMT1. On the contrary, the significant increases in nitrogen, phosphorus and potassium contents following silicon application under salinity stress conditions may be due to the membrane stability by increasing the cell membrane H^±^ATPase activity which induces nutrient uptake, particularly potassium and Ca^+^, however, decreases sodium (Na^+^) uptake and content, thus improving water uptake and photosynthesis. The positive role of silicon in nutrient uptake was reported in some plants [43]. This positive effect of silicon could be due to the role of silicon in decreasing Na+ accumulation in salt-stressed sweet pepper plants. In fact, decreases in both Na^+^ and Cl^-^ levels, however, increase K^+^ [10], in addition to improving water uptake, keeping nutrient balance, and promoting photosynthesis. Silicon can maintain antioxidative capacity, improve osmotic adjustment, and increase the activity of photosynthetic enzymes [47,48].

According to our results in Figure 6, the high level of salinity was more effective than the low level in decreasing the number of fruits and fruit yield. This might be due to the fact that salinity negatively affects and reduces water status and nutrient uptake, leading to a decreased photosynthetic rate, relative water content, and depressed regulation of mainly the zinc finger protein-160. The end results of these processes could be a final reduction in the number of fruits, total fruit yield, and fruit fresh weight. The harmful effect of salinity on growth and production was observed in many plants [3,4,7,47]. In contrast, the obtained data of the present study demonstrate that silicon treatment confers valuable effects on number of fruits, total fruit yield, and fruit fresh weight of sweet pepper under salinity conditions. The valuable effect of silicon may be due to the role of silicon in increasing the expression of RNA polymerase, zinc finger proteins and ribosomal proteins which activates the stress tolerance, increase the soil water holding capacity and enhances photosynthesis as well as reduces transpiration rate [48], and also reduces oxidative stress and improves fruits yield.

Generally, our study revealed that application of silicon on salt-stressed sweet pepper plants alleviates the harmful effects of salinity by enhancing water status, increasing photosynthetic rate, reducing oxidative damage, regulating some osmolytes and phytohormones [49] as well as enhancing antioxidant enzyme activity and, consequently, improving yield production.

## 4. Materials and Methods

### 4.1. Design and Treatments of Experiments

The current research was carried out at Faculty of Agriculture, Kafrelsheikh University, Egypt in the two growth seasons of 2018 and 2019. Tow pot experiments were conducted to examine the influence of silicon on physiological and biochemical characters and fruits yield of sweet pepper plants (*Capsicum annuum* L.) cv. California Wonder under salinity stress at two concentrations (1500 and 3000 ppm) of sodium chloride (NaCl). The laboratory analyses were carried out at the Plant Pathology & Biotechnology Lab and EPECRS Excellence Center, Kafrelsheikh University, Egypt. The seeds were sown in the nursery on the 2nd and 4th of June in the two seasons, respectively. The pots (40 cm diameter) were filled with 8 kg soil and two seedlings, the seedlings were transplanted at forty-two days from sowing. The chemical and physical characters of soil were assayed and the results were as follows: pH 8.2, N 32.3 ppm, P 10.4 ppm, K 290 ppm, electrical conductivity 1.8 dS m^−1^, soil organic matter 1.9%, sand 17.4%, silt 35.6%, and clay 47.3% [50]. The complete fertilizer of nitrogen, phosphorus, and potassium (NPK) (135:40:35 kg ha^−1^) was added in two equal doses as recommendations, the first one after two weeks from transplanting and the second was added at the start of the flowering stage. The plants were irrigated with saline water at two concentrations (1500 and 3000 ppm) and treated with silicon (sodium and potassium silicate at 2.7 mmol L^−1^) twice after 25 and 50 days from transplanting. The experiment was prepared in a completely randomized design with 4 replicates and leaf samples were taken for physiological and biochemical studies at 90 days from transplanting, for the following measurements:

### 4.2. Determination of Chlorophyll a and Chlorophyll b Concentrations

For chlorophyll a and chlorophyll b determination, the samples of sweet pepper fresh leaves, 1 g from each replicate, were taken and placed in 5 mL Dimethyl formamide overnight in the refrigerator. The absorbance was determined spectrophotometrically at 647 and 664 nm by the method of Moran [51].

### 4.3. Relative Water Content (RWC %)

Sweet pepper leaf discs (1 cm in diameter) were taken from the fully expanded leaves. The fresh weight (FW) was recorded, and then the discs were incubated in distilled water for 4 h. Turgid weight was recorded, and the discs were put in an oven at 70 °C for 24 h, to calculate the dry weight (DW). Relative water content was measured by the methodology of Sanchez et al. [52] as follows:

RWC % = (FW − DW)/(TW − DW) × 100, where Fresh weight (FW); dry weight (DW); turgid weight (TW)

### 4.4. Electrolyte Leakage (EL %)

Twenty discs (1 cm^2^) of sweet pepper leaves were put into flasks, each containing 25 mL deionized water. The samples were shaken for 20 h at ambient temperature. First, electrical conductivity (EC1) was recorded for each vial. Flasks were then submerged in a hot water bath at 80 °C for 1 h. The vials were again shaken for 20 h at 21 °C. Last, conductivity (EC2) was measured for each flask. Electrolyte leakage % was calculated as follows: first conductivity/last conductivity × 100. [53].

### 4.5. Lipid Peroxidation (Malondialdehyde)

Lipid peroxidation was determined in fresh leaves as malondialdehyde (MDA) using a spectrophotometer as follow: MDA (nmol g^−1^Fw) = [6.45 × (A532−A600) − (0.56 × A450)] × V^−1^W, where V = volume (ml); W = weight (g). [54]. The leaf extract was mixed with an equal volume of a 0.5% (*w*/*v*) thiobarbituric acid containing 5% (*w*/*v*) trichloroacetic acid, the mixture was heated at 100 °C for 15 min, the absorbance was measured after centrifugation (10 min at 12,000 rpm) at 532, 600, and 450 nm.

### 4.6. Determination of Proline

For proline determination, a solution of glacial acetic acid, proline, and ninhydrin acid (1:1:1) was incubated for 1 h at 90 °C. Then, the samples were cooled in an ice bath. The extraction was done using 2 mL of toluene and the absorbance was determined at 520 nm using a spectrophotometer as μg g^−1^ Fw [55].

### 4.7. Determination of Reactive Oxygen Species (Superoxide and Hydrogen Peroxide)

Hydrogen peroxide and superoxide were calculated by the methodology of Okuda et al. [56] and Elstner and Heupel [57]. Hydrogen peroxide was determined in sweet pepper fresh leaves as follows: 0.5 g pieces of leaves were homogenized in an ice bath with 5 mL of 0.1% (m/v) TCA. The samples were centrifuged for 20 min at 12 000× *g* and 4 °C, 0.5 mL of the supernatant was added to 0.5 mL of a 10 mM potassium phosphate buffer and 1 mL of 1 M KI. The concentration was recorded using a spectrophotometer at 390 nm. Hydrogen peroxide contents were measured using a standard curve according to Okuda et al. [56]. Superoxide was assessed using the method of Elstner and Heupel [58] by observing nitrate formation from hydroxyl amine.

### 4.8. Antioxidant Enzyme Activity

The leaf samples were taken for antioxidant enzyme determination: catalase (CAT), peroxidase (POX) and glutathione reductase (GR) and to assay activities of polyphenol oxidase (PPO). Fresh leaf samples (0.5 g) were homogenized at 0–4 °C in 3 mL of 50 mM TRIS buffer (pH 7.8), containing 1mM EDTA-Na2 and 7.5% polyvinylpyrrolidone. Leaf samples were centrifuged at 12,000 rpm for 20 min and the antioxidant enzyme activity was assayed using a spectrophotometer in the supernatant. Catalase activity (CAT) was determined by the methodology of Aebi [58]. The reaction mixture contained, in a final volume of 2.15 mL, 2 mL 0.1 M Na-phosphate buffer (pH 6.5), 100 μL hydrogen peroxide, and 50 μL leaf extract supernatant. The solution was mixed and the absorbance was recorded at 240 nm for 3 min by a quartz cuvette.

Peroxidase (POX) activity was measured according to the methodology of Hammerschmidt et al. [59]. The mixture consisted of 2.9 mL of a 100 mM sodium phosphate buffer (pH 6.0) containing 0.25% *(v/v)* guaiacol and 100 mM H_2_O_2_. 100 μL of crude enzyme extract was added, the absorbance was recorded every 30 sec at 470 nm for 3 min. The activity of the enzyme was recorded as min^−1^ g^−1^ fresh weight.

The activity of polyphenol oxidase (PPO) was recorded according to Malik and Singh [60]. Glutathione reductase activity was measured by the method of Foyer and Halliwell [61] as unit min^−1^ g^−1^ fresh weight.

### 4.9. Determination of Soluble Sugar, Sucrose and Starch Contents

The fresh leaf samples (0.1 g) were was extracted with ethanol 80% (*v*/*v*) at 80 °C for 30 min and centrifuged for 10 min at 10,000× *g*. The extraction was done three times with 80% ethanol, the supernatants were collected and the final volume adjusted using ethanol to 5 mL. Soluble sugars and sucrose contents were measured by a spectrophotometer at A620 nm and A489 nm, respectively [62]. The extraction of starch was done by evaporation from the residue, distilled water (2 mL) was added to the samples and incubated at 100 °C for 15 min. Starch contents were measured at A620 nm [63].

### 4.10. Determination of Nitrogen, Phosphorus, Potassium, and Sodium

Several minerals (N, P, K, and Na) in sweet pepper leaves were determined. Mixed-acid-digestion was used for mineral extraction using the methodology of Chapman and Parker (1963) [64]. Nitrogen (%) and crude protein (%) was determined using a Kjeldahl method [65]. Colorimetric determination of phosphorus (%) was conducted according to Snell and Snell (1967) [66]. Sodium (Na%) and potassium (K%) contents were determined in digested solutions using a flame photometer according to the methodology of Jakson (1967) [67].

### 4.11. Fruit Yield

At harvest date, the number of fruits plant^−1^, fruit fresh weight per plant (g), and fruit yield (ton hectare^−1^) were determined.

### 4.12. Statistical Analysis

Analysis of variance (ANOVA) procedures was done by the method of Gomez and Gomez (1984) [68] using the MSTAT-C Statistical Software package. The means were compared by Duncan (1955) [69] when the difference was significant (*p* ≤ 0.05).

## 5. Conclusions

We can conclude that salinity stress has a negative impact on sweet pepper plants and this effect can be mitigated by foliar application of silicon. Silicon supplementation was beneficial and plays a pivotal role in alleviating the adverse effects of salinity on sweet pepper growth and fruit yield as well as physiological and biochemical characteristics. Silicon application led to an increased relative water content, concentrations of chlorophyll, activity of antioxidant enzymes and nutrient uptake as well as number of fruits plant^−1^, total fruit yield (ton hectare^−1^), and fruits fresh weight plant^−1^. In contrast, sodium uptake and content, lipid peroxidation and electrolyte leakage were reduced in stressed sweet pepper plants treated with silicon. According to our findings, silicon application led to a reduction of the injurious effects of salinity and enhancement of water status, photosynthetic pigments, activity of antioxidant enzymes, nutrient uptake, and fruit yield of sweet pepper plants. The achieved results from this research will be supportive in enhancing sweet pepper fruit yield during salinity stress in commercial production systems by using silicon treatments.

## References

[1] Yilmaz K., Akinci I.E., Akinci S. (2004). Effect of Salt Stress on Growth and Na, K Contents of Pepper (Capsicum annuum L.) In Germination and Seedling Stages. Pak. J. Biol. Sci..

[2] Majeed A., Muhammad Z., Hasanuzzaman M., Hakeem K.R., Nahar K., Alharby H.F. (2019). Salinity: A Major Agricultural Problem—Causes, Impacts on Crop Productivity and Management Strategies. Plant Abiotic Stress Tolerance.

[3] Abdelaal K.A.A., EL-Maghoursaby L.M., Elansary H., Hafez Y.M., Ibrahim E.I., El-Banna M., El-Esawi M., Elkelish A. (2020). Treatment of Sweet Pepper with Stress Tolerance-Inducing Compounds Alleviates Salinity Stress Oxidative Damage by Mediating the Physio-Biochemical Activities and Antioxidant Systems. Agronomy.

[4] Helaly M.N., Mohammed Z., El-Shaeery N.I., Abdelaal K.A.A., Nofal I.E. (2017). Cucumber grafting onto pumpkin can represent an interesting tool to minimize salinity stress. Physiological and anatomical studies. Middle East J. Agric. Res..

[5] Kosova K., Vıtamvas P., Prasil I.T., Renaut J. (2011). Plant proteome changes under abiotic stress-contribution of proteomics studies to understanding plant stress response. J. Proteom..

[6] Allakhverdiev S.I., Sakamoto A., Nishiyama Y., Inaba M., Murata N. (2000). Ionic and osmotic effects of NaCl-induced inactivation of photosystems I and II *Synechococcus* sp.. Plant Physiol..

[7] Hasan M.K., El Sabagh A., Sikdar M.S., Alam M.J., Ratnasekera D., Barutcular C., Abdelaal K.A., Islam M.S. (2017). Comparative adaptable agronomic traits of blackgram and mungbean for saline lands. Plant Arch..

[8] Agarie S., Hanaoka N., Ueno O. (1998). Effects of silicon on tolerance to water deficit and heat stress in rice plants (*Oryza sativa* L.), monitored by electrolyte leakage. Plant Prod. Sci..

[9] Zhu Z., Wei G., Li J., Qian Q., Yu J. (2004). Silicon alleviates salt stress and increases antioxidant enzymes activity in leaves of salt-stressed cucumber (*Cucumis sativus* L.). Plant Sci..

[10] Liang Y.C., Ding R.X. (2002). Influence of silicon on microdistribution of mineral ions in roots of salt-stressed barley as associated with salt tolerance in plants. Sci. China Ser. C.

[11] Abdelaal K.A.A. (2015). Effect of Salicylic acid and Abscisic acid on morpho-physiological and anatomical characters of faba bean plants (*Vicia faba* L.) under drought stress. J. Plant Prod. Mansoura Univ..

[12] Abdelaal K.A.A., Hafez Y.M., El-Afry M., Tantawy D.S., Alshaal T. (2018). Effect of some osmoregulators on photosynthesis, lipid peroxidation, antioxidative capacity, and productivity of barley (*Hordeum vulgare* L.) under water deficit stress. Environ. Sci. Pollut. Res..

[13] Abdelaal K.A.A., Attia K.A., Alamery S.F., El-Afry M., Ghazy A., Tantawy D., Al-Doss A., El-Shawy E.-S.E., Abu-Elsaoud A., Hafez Y.M. (2020). Exogenous Application of Proline and Salicylic Acid can Mitigate the Injurious Impacts of Drought Stress on Barley Plants Associated with Physiological and Histological Characters. Sustainability.

[14] Elkelish A., Qari S.H., Mazrou Y.M., Abdelaal K.A.A., Hafez Y.M., Abu-Elsaoud A.M., Batiha G., El-Esawi M., El Nahhas N. (2020). Exogenous Ascorbic Acid Induced Chilling Tolerance in Tomato Plants Through Modulating Metabolism, Osmolytes, Antioxidants, and Transcriptional Regulation of Catalase and Heat Shock Proteins. Plants.

[15] Esmail S.M., Omara R.I., Abdelaal K.A.A., Hafez Y.M. (2019). Histological and biochemical aspects of compatible and incompatible wheat-*Puccinia striiformis* interactions *Physiol*. Mol. Plant Pathol..

[16] Morton M.J., Awlia M., Al-Tamimi N., Saade S., Pailles Y., Negrão S., Tester M. (2019). Salt stress under the scalpel–dissecting the genetics of salt tolerance. Plant J..

[17] Al Mahmud J., Bhuyan M.H.M.B., Anee T.I., Nahar K., Fujita M., Hasanuzzaman M., Hasanuzzaman M., Hakeem K.R., Nahar K., Alharby H.F. (2019). Reactive Oxygen Species Metabolism and Antioxidant Defense in Plants under Metal/Metalloid Stress. Plant Abiotic Stress Tolerance.

[18] Boling L., Prabhakaran S., Abinaya M. (2019). Mechanisms of Silicon-Mediated Amelioration of Salt Stress in Plants. Plants.

[19] Rao S., Du C., Li A., Xia X., Yin W., Chen J. (2019). Salicylic Acid Alleviated Salt Damage of Populus euphoursatica: A Physiological and Transcriptomic Analysis. Forests.

[20] Conceição S., Neto C., Marques E. (2019). Silicon modulates the activity of antioxidant enzymes and nitrogen compounds in sunflower plants under salt stress. Arch. Agron. Soil Sci..

[21] Calero A., Aparecida D., Prado R. (2019). Silicon attenuates sodium toxicity by improving nutritional efficiency in sorghum and sunflower plants. Plant Physiol. Biochem..

[22] Soundararajan P., Sivanesan I., Jana S., Jeong B.R. (2014). Influence of silicon supplementation on the growth and tolerance to high temperature in Salvia splendens. Hortic. Environ. Biotechnol..

[23] Li H., Zhu Y., Hu Y., Han W., Gong H. (2015). Beneficial effects of silicon in alleviating salinity stress of tomato seedlings grown under sand culture. Acta Physiol. Plant..

[24] Luyckx M., Hausman J.F., Lutts S., Guerriero G. (2017). Silicon and plants: Current knowledge and technological perspectives. Front. Plant Sci..

[25] Debona D., Rodrigues F.A., Datnoff L.E. (2017). Silicon’s role in abiotic and biotic plant stresses. Annu. Rev. Phytopathol..

[26] Etesami H., Jeong B.R. (2018). Silicon (Si): Review and future prospects on the action mechanisms in alleviating biotic and abiotic stresses in plants. Ecotoxicol. Environ. Saf..

[27] Yin L., Wang S., Li J., Tanaka K., Oka M. (2013). Application of silicon improves salt tolerance through ameliorating osmotic and ionic stresses in the seedling of Sorghum bicolor. Acta Physiol. Plant..

[28] Manivannan A., Soundararajan P., Muneer S., Ko C.H., Jeong B.R. (2016). Silicon mitigates salinity stress by regulating the physiology, antioxidant enzyme activities, and protein expression in *Capsicum annuum* ‘Bugwang’. BioMed Res. Int..

[29] Huang X.Y., Chao D.Y., Gao J.P., Zhu M.Z., Shi M., Lin H.X. (2009). A previously unknown zinc finger protein, DST, regulates drought and salt tolerance in rice via stomatal aperture control. Genes Dev..

[30] Muneer S., Park Y.G., Manivannan A., Soundararajan P., Jeong B.R. (2014). Physiological and proteomic analysis in chloroplasts of *Solanum lycopersicum* L. under silicon efficiency and salinity stress. Int. J. Mol. Sci..

[31] Li J., Hu L., Zhang L., Pan X., Hu X. (2015). Exogenous spermidine is enhancing tomato tolerance to salinity–alkalinity stress by regulating chloroplast antioxidant system and chlorophyll metabolism. BMC Plant Biol..

[32] Dawood M.G., Taie H.A.A., Nassar R.M.A., Abdelhamid M.T., Schmidhalter U. (2014). The changes induced in the physiological, biochemical and anatomical characteristics of *Vicia faba* by the exogenous application of proline under seawater stress. S. Afr. J. Bot..

[33] Sharma S., Villamor J.G., Verslues P.E. (2011). Essential role of tissue-specific proline synthesis and catabolism in growth and redox balance at low water potential. Plant Physiol..

[34] Shahid M., Balal R., Pervez M., Abbas T., Aqeel M., Javaid M., Garcia-Sanchez F. (2015). Foliar spray of phyto-extracts supplemented with silicon: An efficacious strategy to alleviate the salinity-induced deleterious effects in pea (*Pisum sativum* L.). Turk. J. Bot..

[35] Khoshgoftarmanesh A.H., Khodarahmi S., Haghighi M. (2014). Effect of silicon nutrition on lipid peroxidation and antioxidant response of cucumber plants exposed to salinity stress. Arch. Agron. Soil Sci..

[36] Alsaeedi A., El-Ramady H., Alshaal T., El-Garawany M., Elhawat N., Al-Otaibi A. (2019). Silica nanoparticles boost growth and productivity of cucumber under water deficit and salinity stresses by balancing nutrients uptake. Plant Physiol. Biochem..

[37] Shah S., Houborg R., McCabe M. (2017). Response of Chlorophyll, Carotenoid and SPAD-502 Measurement to Salinity and Nutrient Stress in Wheat (*Triticum aestivum* L.). Agronomy.

[38] El-Banna M.F., Abdelaal K.A.A. (2018). Response of Strawberry Plants Grown in the Hydroponic System to Pretreatment with H2O2 before Exposure to Salinity Stress. J. Plant Prod..

[39] Wang Y., Li X., Li J., Bao Q., Zhang F., Tulaxi G., Wang Z. (2016). Salt-induced hydrogen peroxide is involved in modulation of antioxidant enzymes in cotton. Crop J..

[40] Lin C.C., Kao C.H. (2000). Effect of NaCl stress on H2O2 metabolism in rice leaves. Plant Growth Regul..

[41] Hernandez M., Fernandez-Garcia N., Diaz-Vivancos P., Olmos E. (2010). A different role for hydrogen peroxide and the antioxidative system under short and long salt stress in *Brassica oleracea* roots. J. Exp. Bot..

[42] Li Q., Lv L.R., Teng Y.J., Si L.B., Ma T., Yang Y.L. (2018). Apoplastic hydrogen peroxide and superoxide anion exhibited different regulatory functions in salt-induced oxidative stress in wheat leaves. Biol. Plant..

[43] Vighi I.L., Benitez L.C., Amaral M.N., Moraes G.P., Auler P.A., Rodrigues G.S., Deuner S., Maia L.C., Braga E.J.B. (2017). Functional characterization of the antioxidant enzymes in rice plants exposed to salinity stress. Biol. Plant..

[44] Omara R.I., Abdelaal K.A.A. (2018). Biochemical, histopathological and genetic analysis associated with leaf rust infection in wheat plants (*Triticum aestivum* L.). Physiol. Mol. Plant Pathol..

[45] Đorđević N.O., Todorović N., Novaković I.T., Pezo L.L., Pejin B., Maraš V., Tešević V.V., Pajović S.B. (2018). Antioxidant Activity of Selected Polyphenolics in Yeast Cells: The Case Study of Montenegrin Merlot Wine. Molecules.

[46] Wani S.H., Brajendra Singh N., Haribhushan A., Iqbal Mir J. (2013). Compatible Solute Engineering in Plants for Abiotic Stress Tolerance—Role of Glycine Betaine. Curr. Genom..

[47] Liu W., Zhang Y., Yuan X., Xuan Y., Gao Y., Yan Y. (2016). Exogenous salicylic acid improves salinity tolerance of Nitraria tangutorum. Russ. J. Plant Physiol..

[48] Zhu Y., Gong H. (2014). Beneficial effects of silicon on salt and drought tolerance in plants. Agron. Sustain. Dev..

[49] Jeong M.J., Park S.C., Byun M.O. (2001). Improvement of salt tolerance in transgenic potato plants by glyceraldehyde-3 phosphate dehydrogenase gene transfer. Mol. Cells.

[50] Page A.L., Miller R.H., Keeney D.R. (1982). Methods of Soil Analysis.

[51] Moran R. (1982). Formulae for Determination of Chlorophyllous Pigments Extracted with N,N-Dimethylformamide 1. Plant Physiol..

[52] Sanchez F.J., de Andrés E.F., Tenorio J.L., Ayerbe L. (2004). Growth of epicotyls, turgor maintenance and osmotic adjustment in pea plants (*Pisum sativum* L.) subjected to water stress. Field Crop. Res..

[53] Dionisio-Sese M.L., Tobita S. (1998). Antioxidant responses of rice seedlings to salinity stress. Plant Sci..

[54] Davenport S.B., Gallego S.M., Benavides M.P., Tomaro M.L. (2003). Behaviour of antioxidant defense system in the adaptive response to salt stress in *Helianthus annuus* L. cells. Plant Growth Regul..

[55] Bates L.S., Waldren R.P., Teare I.D. (1973). Rapid determination of free proline for water-stress studies. Plant Soil.

[56] Okuda T., Masuda Y., Yamanka A., Sagisaka S. (1991). Abrupt increase in the level of hydrogen peroxide in leaves of winter wheat is caused by cold treatment. Plant Physiol..

[57] Elstner E.F., Heupel A. (1976). Inhibition of nitrite formation from hydroxylammonium chloride: A simple assay for superoxide dismutase. Anal. Biochem..

[58] Aebi H. (1984). Catalase in vitro. Methods in Enzymology; Oxygen Radicals in Biological Systems.

[59] Hammerschmidt R., Nuckles E.M., Kuć J. (1982). Association of enhanced peroxidase activity with induced systemic resistance of cucumber to *Colletotrichum lagenarium*. Physiol. Plant Pathol..

[60] Malik C.P., Singh M.B. (1980). Plant Enzymology and Histo-Enzymology.

[61] Halliwell B., Foyer C.H. (1978). Properties and Physiological Function of a Glutathione Reductase Purified from Spinach Leaves by Affinity Choursomatography. Planta.

[62] Xu W., Cui K., Xu A., Nie L., Huang J., Peng S. (2015). Drought stress condition increases root to shoot ratio via alteration of carbohydrate partitioning and enzymatic activity in rice seedlings. Acta Physiol. Plant..

[63] Kuai J., Liu Z., Wang Y., Meng Y., Chen B., Zhao W., Zhou Z., Oosterhuis D.M. (2014). Waterlogging during flowering and boll forming stages affects sucrose metabolism in the leaves subtending the cotton boll and its relationship with boll weight. Plant Sci..

[64] Chapman H.D., Parker F.P. (1963). Methods of Analysis for Soils, Plants and Waters.

[65] Barbano D.M., Lynch J.M. (1990). Kjeldahl Method for Determination of Total Nitrogen Content of Milk, Collaborative Study. J. Assoc. Off. Anal. Chem..

[66] Snell F.D., Snell C.T. (1976). Colorimetric Methods of Analysis.

[67] Jakson M.L. (1976). Soil Chemical Analysis, Prentice Hall of India Private Limited.

[68] Gomez K.A., Gomez A.A. (1984). Statistical Procedures for Agricultural Research.

[69] Duncan B.D. (1955). Multiple ranges and multiple F-test. Biometria.

